# Mixing and diffusion in a two-type population

**DOI:** 10.1098/rsos.172102

**Published:** 2018-02-21

**Authors:** Segismundo S. Izquierdo, Luis R. Izquierdo, Dunia López-Pintado

**Affiliations:** 1 Department of Industrial Organization, EII, Universidad de Valladolid, paseo del cauce 59, 47011 Valladolid, Spain; 2 Department of Civil Engineering, Universidad de Burgos, Edificio A, Avda. Cantabria s/n, 09006 Burgos, Spain; 3 Department of Economics, Universidad Pablo de Olavide, Carretera de Utrera, Km. 1, 41013 Sevilla, Spain; 4 Center for Operations Research and Econometrics, Voie du Roman Pays, 34, 13848 Louvain-la-Neuve, Belgium

**Keywords:** diffusion, mixing, segregation, homophily, SIS

## Abstract

The outbreak of epidemics, the rise of religious radicalization or the motivational influence of fellow students in classrooms are some of the issues that can be described as diffusion processes in heterogeneous groups. Understanding the role that interaction patterns between groups (e.g. homophily or segregation) play in the diffusion of certain traits or behaviours is a major challenge for contemporary societies. Here, we study the impact on diffusion processes of mixing (or, alternatively, segregating) two groups that present different sensitivities or propensities to contagion. We find non-monotonic effects of mixing and inefficient segregation levels, i.e. situations where a change in the mixing level can benefit both groups, e.g. where an increase in the mixing level can reduce the expected contagion levels in both groups. These findings can have fundamental consequences for the design of inclusion policies.

## 1. Introduction

We consider a contagion process [1] in a population made up by two groups, where each group presents a different contagion propensity. Individuals regularly interact with other individuals, who may belong to the same group (intra-group interactions) or to the other group (between-group interactions). The mixing level is measured by the fraction of between-group interactions. In this paper, we study how mixing—or, alternatively, segregating—the two groups may affect the expected level of diffusion of a certain trait. We show that a particularly interesting case may arise, where a change in the mixing level can improve the well-being of both groups in the population, i.e. where there are *Pareto-inefficient mixing levels* [2]. In the case of a disease, this would imply that increasing the mixing between the two groups can reduce the infection levels in both groups.

The first model we analyse is a multi-type susceptible–infected–susceptible (SIS) model [3,4] with two groups, where each group presents a different propensity to contagion. Initially, we assume that all individuals within the same group present the same susceptibility to contagion, but this hypothesis is relaxed later. Depending on parameter values, the multi-type SIS process has been shown to converge either (i) from any initial conditions, to the situation where there is no diffusion in either group, or (ii) from any *non-null* initial fraction of infected individuals, to a positive *almost globally asymptotically stable state* [3,4]. Here we investigate the impact of mixing the two groups on their respective infection levels at the positive stable state. We also explore the robustness of our results to more general settings.

Among the most related papers in the literature^1^, [3,4] analyse the existence of an almost globally asymptotically stable state (the endemic state [9,10]) in the multi-type SIS model, and their results constitute the starting point for our study of the effect of mixing on such state for the two-type SIS case. An extension of the analysis in [3,4] to varying population size can be found in [11]. Epidemic thresholds [12] for a multi-type contagion model with different mixing levels are studied in [13]. Their framework includes the SIS model as a particular case, so their analysis of the local stability of the no-infection state applies to the two-type SIS model too. Epidemic thresholds and endemic equilibria in a two-type SIS model considering a bipartite network (which corresponds to the highest possible level of mixing in our setting) are studied in [14,15], while the analysis in [16] deals with random mixing without bias. The models in [13–16] generalize our approach by considering different interaction frequencies or network degree distributions, but the analysis of the endemic state in those papers corresponds to one particular mixing value (either a bipartite network [14,15] or random mixing [16]), while here we consider arbitrary mixing levels and explore the sensitivity to mixing of the endemic state.^2^

**Figure 1.**
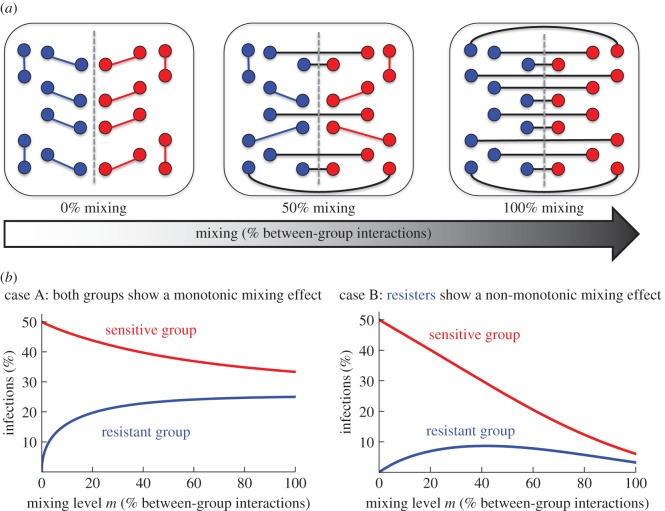
Interaction structure and infection levels as a function of mixing. (*a*) (From left to right) Examples of a segregated population case (*m* = 0), an unbiasedly mixed population case (*m* = 0.5) and a bipartite population case (*m* = 1). (*b*) Infection levels in equilibrium for the resistant group (blue) and the sensitive group (red) as a function of the mixing level *m* for two different cases. In case B, the infection level in the resistant group is a non-monotonic function of the mixing level, and Pareto inefficient mixing levels exist. Parameters {λ_1_, λ_2_} for the SIS model: case A: {1, 2}, case B: {0.55, 2}.

As a first illustration of our main results, consider the case of an infectious disease spreading in a population composed of two distinct groups of individuals (figure 1): one group is more sensitive to contagion (the *sensitive group*, in red) and the other group is less sensitive (the *resistant group*, in blue). Note that the two groups need not be defined by their contagion propensities: some other variable, such as race, the presence of some gene or habitat, could characterize the partition of the population into two groups. If the group-defining variable is somewhat correlated with the sensitivity to contagion, then the two groups will present different average sensitivities, which is the only condition required in our model.

We are interested in analysing the consequences of changing the level of mixing between groups. To conduct such an analysis in the simplest possible way, we keep the average frequency of individual interactions constant, and we modify the fraction of interactions between individuals belonging to different groups. One might intuitively predict that, as the level of mixing increases, the infection levels in both groups should approximate, with an increase of infections in the resistant group and a decrease of infections in the sensitive group. Thus, the sensitive group would always benefit from a higher fraction of between-group interactions, whereas the resistant group would always be harmed by it.^3^

We show that, while the first part of the previous intuition holds true (i.e. the infection levels do approach as mixing is increased), the second part is not always true, i.e. the resistant group may benefit from interacting more with the sensitive group. In other words, there can be *inefficient mixing levels*, i.e. situations where increasing between-group interactions leads to a reduction of infections in *both* groups (figure 1*b*, case B).

The underlying reason for this paradoxical effect is the feedback loop created between groups: from an initial stable situation corresponding to a given mixing level (e.g. 60% mixing in the scenario corresponding to case B in figure 1*b*), increasing the interaction level between groups can be initially costly for the resistant group, which will initially meet more infected individuals. However, it can turn out to be beneficial for that same group once the returns from the positive effect induced on the other group are collected (i.e. the reduction in the infection level of the sensitive group), and a new equilibrium is obtained in which both groups are better off.

The infection narrative constitutes a natural motivating example, but the framework can be extended to a variety of contexts [1,20–22]. In the infection narrative, individuals may be in an infected or in a susceptible state; in other contexts, the state of infection can be interpreted as the state of adopting a particular behaviour, technology, idea or belief, versus the state of adopting another alternative. In these cases, we will use the more general term *diffusion level*, instead of *infection level*, for the fraction of individuals in a group adopting one of the states.

In the social networks literature, the question we address can be more naturally posed as: ‘how do different levels of homophily or segregation affect the diffusion of a trait?’, where ‘homophily’ refers to the tendency of individuals in one group to interact preferentially with other individuals in the same group [23]. This issue is relevant because many social interactions exhibit significant homophily based on characteristics such as race, age, profession, etc. [24,25], while some other relationships (i.e. buyer-seller networks) are characterized by a high degree of heterophily or disassortative mixing.

In a practical situation, regardless of the existing level of mixing, a decision maker or social planner could potentially influence the interaction patterns between groups by implementing specific incentives, rules or goal-directed policies. The key question, then, is how induced changes in between-group interaction patterns affect diffusion. Examples include healthcare programmes to promote interactions between individuals of similar health characteristics [26], or compositional classroom designs to encourage school students with high academic motivation to interact with students with lower motivation [27–29].^4^

The rest of the paper is organized as follows. In §2 we present the model. Section 3 contains the formal results. In §4, motivated by some of the potential fields of application, we present and analyse a generalization of the SIS model. Finally, §5 presents a discussion and the concluding remarks. The formal proofs and a robustness analysis can be found in the electronic supplementary material.

## 2. Two-type SIS model

Our model is based on the susceptible–infected–susceptible (SIS) contagion framework, extended to a multi-type setting [3]. In particular, we focus on a population composed of two groups of equal size: one of the groups is the resistant group and the other one is the sensitive group. Individuals can be in one of two possible states: ‘susceptible’ or ‘infected’. In each time period, each individual interacts with another individual with probability *p* > 0 and, depending on the state of its partner, may become infected. Specifically, a susceptible individual in group *i* ∈ {1, 2} becomes infected with probability *υ_i_* > 0 if it happens to interact with an infected individual. Individuals who are already infected recover and become susceptible again with probability *δ_i_* > 0.

Let *m* ∈ [0, 1] represent the mixing level, i.e. the probability that an interacting individual of one particular group meets an individual from the other group. Thus, *m* is the expected fraction of between-group interactions. As illustrated in figure 1, if *m* = 0, individuals interact only with individuals from their own group (which is the fully segregated case), whereas if *m* = 1, individuals interact only with individuals from the other group (which is the bipartite case).^5^

We use a continuous-time mean-dynamic approximation to study the evolution of the adoption levels in each group. To do so, let *ρ_i_* denote the fraction of infected individuals in group *i*. The evolution of *ρ_i_* in each group over time is described by the following nonlinear system of differential equations:

2.1 $${\dot{\rho }}_{i}=p\upsilon_{i}(1-\rho_{i})[m\rho_{j}+(1-m)\rho_{i}]-\delta_{i}\rho_{i},$$

where *m* is the mixing level, *i*, *j* ∈{1, 2} and *i *≠ *j*.

To interpret these equations, note that, in order to get infected, a non-infected individual in group *i*—whose number is proportional to $\left(1-\rho_{i}\right)$—must interact with an infected individual of either the same or the other group—which happens with probability $p[m\rho_{j}+(1-m)\rho_{i}]$—and get infected—which then happens with conditional probability $\upsilon_{i}$. In turn, the number of recoveries per period is proportional to the number of infected individuals—whose prevalence is *ρ_i_*—and to the probability of recovery *δ_i_*.

Note that parameters *p*, *υ_i_* and *δ_i_* can be interpreted as rates instead of probabilities. We can rewrite these equations as

2.2 $${\dot{\rho }}_{i}=\delta_{i}[\lambda_{i}(1-\rho_{i})[m\rho_{j}+(1-m)\rho_{i}]-\rho_{i}],$$

where λ*_i_* = (*p υ_i_*/*δ_i_*) represents the *effective adoption rate for group i.* Thus, the equilibria and the long-run dynamics of this model can be characterized using exclusively three parameters: λ_1_, λ_2_ and *m*.

The SIS equations analysed in the paper constitute the deterministic mean-dynamic [33] of the described underlying stochastic process. These equations provide a good approximation to the actual stochastic dynamics occurring in finite populations when those populations are large enough [33,34]. In the electronic supplementary material, we show the robustness of the reported results to different population sizes, as well as to the inclusion of some heterogeneity within groups.

## 3. Formal results

**Figure 2.**
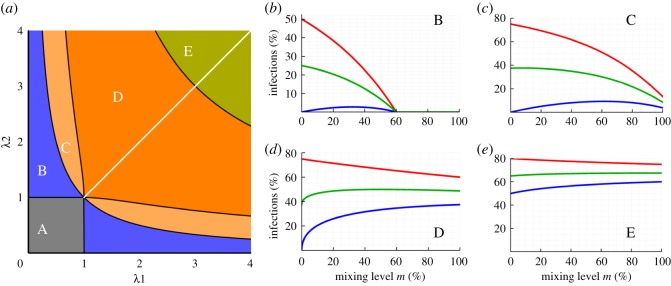
Regions of effective adoption rate values (λ_1_ and λ_2_) corresponding to qualitatively different effects of the mixing level *m* on equilibrium infection levels. The main graph (*a*) illustrates the different regions in the {λ_1_, λ_2_} plane. The accompanying graphs (*b*–*e*) represent the stable equilibrium infection levels (blue for the resistant group, red for the sensitive group, and green for the average) as functions of *m* for {λ_1_, λ_2_} values corresponding to regions B, C, D and E, respectively. In region A, there is no diffusion. The {λ_1_, λ_2_} values selected for the graphs are: B: {0.25, 2}, C: {0.3, 4}, D: {1, 4}, and E: {2, 5}. An interactive program to check the effect of mixing for different adoption rates is available at http://demonstrations.wolfram.com/MixingAndInfectionInATwoGroupSISModel/.

In this section, we state the results obtained from the analysis of the model. Detailed proofs of the propositions can be found in the electronic supplementary material. The results in this section are graphically summarized in figure 2, where the space of plausible effective adoption rates (λ_1_, λ_2_) ∈ [0, ∞) × [0, ∞) has been partitioned into different regions according to the impact of the mixing level *m* on the equilibrium diffusion values in each group, and on the population average.

In region A, the no-diffusion state is a global attractor. In region B, there is a positive threshold $\bar{m}<1\;$ such that for $0<m<\bar{m}$ there is a positive almost globally asymptotically stable state (leading to positive diffusion in both groups), while for $m>\;\bar{m}$ the dynamics converge to the no-diffusion state. In the remaining regions, for any positive value of the mixing level *m* and any initial state with some infection*,* the process converges to a positive almost globally asymptotically stable state.

In regions B and C, the infection level for the resistant group is a non-monotonic function of *m*, with an interior maximum. In region B, the average infection level is monotonic decreasing. In regions C and D, the average infection level (*ρ*_1_ + *ρ*_2_)/2 is a non-monotonic function of *m*, with an interior maximum. Finally, in region E, the three infection levels are monotonic functions of *m*.

For the formal propositions, and without loss of generality, let us assume that group 2 is the sensitive group, i.e. 0 < λ_1_ < λ_2_. We are interested in the stationary states and the dynamics of equation (2.2) for m∈(0, 1); the extreme cases *m* = 0, *m* = 1 and λ_1_ = λ_2_ are discussed in the electronic supplementary material.

The stationary states of equation (2.2) are the pairs of values $(\rho_{1},\rho_{2})\in {\left[0,\;1\right]}^{2}$ such that ${\dot{\rho }}_{1}=0$ and ${\dot{\rho }}_{2}=0$. Given the specification of the model, which precludes spontaneous infection, the state where all individuals are susceptible (i.e. no individual is infected) is always stationary. Depending on the values of the parameters, the diffusion dynamics either converge from any initial state to the situation where there is no diffusion in either group, or present a positive *almost globally asymptotically stable state* to which the system converges from any *non-null* initial fraction of infections. The following result formalizes this statement.

**Proposition 3.1 (Globally asymptotically stable state).**

*If* λ_2_ > 1 *and either* λ_1_λ_2_ > 1 *or* $m<\bar{m}$, *where* $\bar{m}=1-(1-\lambda_{1}\lambda_{2})/(\lambda_{2}+\lambda_{1}-2\lambda_{1}\lambda_{2})$*, there is a positive globally asymptotically stable state in* ${\left[0,\;1\right]}^{2}\setminus \{(0,\;0)\}$*. Otherwise the no-diffusion state* (0, 0) *is globally asymptotically stable in* [0, 1]^2^.

As an immediate corollary of proposition 3.1, if the effective adoption rate in the sensitive group is not greater than one (λ_2_ ≤ 1), then the process converges to the no-diffusion state (0, 0), which is globally asymptotically stable. The interesting cases, consequently, are those in which the effective adoption rate in at least one of the groups is greater than one.

The threshold $\bar{m}$ coincides, naturally, with the local-stability threshold of the no-diffusion state characterized in [13] for this case. Proposition 3.1 can be derived, after some adaptations, from the results in [3] or [4] for multi-type SIS models. In the electronic supplementary material, we present an alternative proof for the two-type case that is helpful to show equivalent results for some parametrizations of the general contagion model that we analyse later.

Given that there is a unique globally asymptotically stable state in our dynamics, we can proceed by conducting comparative static analyses. In particular, we can study the effect of the mixing level *m* on the stable infection levels for the sensitive and resistant groups separately, within the range of positive values of *m* where the stable positive equilibrium exists, i.e. for λ_2_ > 1, m∈(0, 1] if λ_1_λ_2_ > 1 and $m\in (0,\;\bar{m})$ if $\lambda_{1}\lambda_{2}\leq 1$. Let $\rho_{i}^{E}$ denote the fraction of infected individuals in group *i* at the positive stable equilibrium (which is a function of *m*). By analysing the derivatives of $\rho_{i}^{E}$ with respect to *m* in the considered range of values where the positive equilibrium exists, we obtain the following results for the positive equilibrium infection levels.

**Proposition 3.2 (Infection level in the sensitive group).**

*The stable positive equilibrium infection level in the sensitive group* $\rho_{2}^{E}$ *is a strictly decreasing function of the mixing level m. Its maximum level,* $1-\lambda_{2}^{-1}$*, corresponds to m* = 0*. If* $\lambda_{1}\lambda_{2}\leq 1$*, then* $\rho_{2}^{E}$ *decreases to 0 as* $m\to {\bar{m}}^{-}$*, with* $\bar{m}\leq 1$*. If* $\lambda_{1}\lambda_{2}>1$*,* $\rho_{2}^{E}$ *obtains its minimum value* $(\lambda_{1}\lambda_{2}-1)/(\;\lambda_{1}\lambda_{2}+\lambda_{1})>0$ *at m* = 1.

Proposition 3.2 implies that, by increasing the mixing level, the infection level in the sensitive group can only decrease (figure 2). If being infected is undesirable, the sensitive group can only benefit from more mixing. Alternatively, if being infected is desirable, the sensitive group can only be harmed by more mixing. Note that if the resistant group is resistant enough (specifically, if $\lambda_{1}<\lambda_{2}^{-1}$, with λ_2_ > 1), then a sufficiently high level of mixing would kill the infection in the whole population. This could be either desirable or undesirable, as it can mean the disappearance of a disease, but it can also mean the disappearance of a desirable trait or behaviour from the whole population.

**Proposition 3.3 (Infection level in the resistant group).**

(*i*) For adoption rates $\lambda_{1}>\sqrt{\lambda_{2}}/(\lambda_{2}-\sqrt{\lambda_{2}}+1)$, which is always the case if λ_1_ > 1, the stable positive infection level in the resistant group $\rho_{1}^{E}$ is a strictly increasing function of the mixing level m∈(0, 1). The minimum level for $\rho_{1}^{E}$ is $\lim_{m\to 0^{+}}\rho_{1}^{E}=\mathrm{Max}(0,1-\lambda_{1}^{-1})$, corresponding to *m*  =  0.
(*ii*) For adoption rates $\lambda_{1}<\sqrt{\lambda_{2}}/(\lambda_{2}-\sqrt{\lambda_{2}}+1)$, the stable positive infection level in the resistant group $\rho_{1}^{E}$ is a non-monotonic function of m which increases from $\lim_{m\to 0^{+}}\rho_{1}^{E}=0$, obtains a maximum value at some interior value of m and then decreases, either back to $\lim_{m\to {\bar{m}}^{-}}\rho_{1}^{E}=0$ if $\lambda_{1}\lambda_{2}\leq 1$, or to $(\lambda_{1}\lambda_{2}-1)\text{/( }\lambda_{1}\lambda_{2}+\lambda_{2})>0$ (obtained at *m*  =  1) if $\lambda_{1}\lambda_{2}>1.$

Proposition 3.3 implies that, if the effective adoption rate in the resistant group is greater than one (meaning that both groups, if isolated, would sustain some infection in equilibrium), then, by increasing the mixing level, the infection level in the sensitive group decreases and the infection level in the resistant group increases. In this case, favouring one group by changing the mixing level can only harm the other.^6^ This behaviour corresponds to parameter regions D and E in figure 2, which are defined by the first condition in proposition 3.3 (which also assumes λ_1_ < λ_2_ for the sake of notational simplicity and without loss of generality).

More interesting findings are obtained in regions B and C in figure 2, wherein the effective adoption rate is greater than one in the sensitive group but lower than one in the resistant group.^7^ In these regions, corresponding to the second part of proposition 3.3, the equilibrium infection level in the resistant group is null when the group is isolated; it increases initially with more mixing and eventually decreases (possibly back to zero again, if in region B) after having obtained some interior maximum value. This implies that there are inefficient mixing levels regardless of whether being infected is desirable or undesirable, as we explain below.

If being infected is desirable, an example of an inefficient mixing level would be the bipartite situation (*m* = 1) in graph C of figure 2, where both groups would increase their equilibrium infection level by reducing the mixing level to around 70%. Again in graph C, if being infected is undesirable, all mixing levels for which the infection level in the resistant group is greater than the value corresponding to the bipartite situation (*m* = 1) would be inefficient, because, by increasing the mixing level to *m* = 1, both groups would decrease their equilibrium infection level. Finally, in region B (where $\lambda_{1}\lambda_{2}\leq 1$), for sufficiently high mixing levels, the infection would even be eliminated from the whole population.^8^

The following proposition concerns the average infection level in the population at the stable positive equilibrium $\rho^{E}=(\rho_{1}^{E}+\rho_{2}^{E})/2$, which is a function of *m* in the range where the positive equilibrium exists.

**Proposition 3.4 (Average infection level).**

(*i*) *If* $\lambda_{1}\lambda_{2}\leq 1$*, the average infection level* $\rho^{E}$ *is a decreasing function of m, with* $\lim_{m\to {\bar{m}}^{-}}\rho^{E}=0$.
(*ii*) *If* $\lambda_{1}^{3/4}(\lambda_{2}+1)\geq \lambda_{2}^{3/4}(\lambda_{1}+1)$*, which requires* λ_1_λ_2_ > 1 *and* λ_2_ > 3, $\rho^{E}$ *is an increasing function of m*.
(*iii*) *Otherwise* $\rho^{E}$ *is a non-monotonic function of m, initially increasing and then decreasing, which obtains an interior maximum. In this case, the minimum average diffusion is obtained either at m* = 1, *if* $\lambda_{1}<\sqrt{\lambda_{2}}\text{/}(\lambda_{2}-\sqrt{\lambda_{2}}+1)$*, or at m* = 0 *otherwise*.

Proposition 3.4 implies that the average infection level decreases to zero in region B, is non-monotonic in regions C and D, and is increasing in region E. This has relevant implications as to the optimal mixing level when the objective is maximizing or minimizing the average infection level. In particular, if the adoption rates lie in region B, then average diffusion is maximized in the segregated case (*m* = 0) and minimized (reaching no diffusion) whenever the mixing level is sufficiently high (i.e. $m>\;\bar{m}$). If the adoption rates lie in regions C or D, average diffusion is maximized at an intermediate level of mixing, and minimized at one of the extremes (*m* = 1 in region C, *m* = 0 in region D). Finally, if the adoption rates lie in region E, average diffusion is an increasing function of mixing, so it is maximized in the bipartite case (*m* = 1) and minimized in the segregated case (*m* = 0).

**Proposition 3.5 (Difference between infection levels).**

*The difference between the stable positive infection levels in the sensitive and resistant groups* $\left(\rho_{2}^{E}-\rho_{1}^{E}\right)$ *is a strictly decreasing function of the mixing level m. This difference is always positive for* $\left(\rho_{1}^{E},\rho_{2}^{E})\neq (0,0\right)$.

Proposition 3.5 implies that the difference between the infection levels of both groups is always minimized at the bipartite situation (*m* = 1).

## 4. General contagion model

The SIS model of contagion analysed in the previous sections can be extended to address more general situations [1,20–22,35], such as peer effects in classrooms, where it seems reasonable to assume that the probability of switching from infected (i.e. motivated student) to susceptible (i.e. non-motivated student) is affected by the current state of partners. A similar issue is how the internal organization of a work team, where some individuals are easier to motivate than others, affects the overall level of productivity. On the one hand, in these cases, as in the SIS model, an important factor for an individual to switch from one state to the other can be the state of his/her partner. On the other hand, there are some limitations of the SIS model when considering social contagion applications [36]: in a SIS model only one of the states is contagious, while the recovery rate is independent of whether one's partner is infected or not. Here we consider a more general framework which, even though it might still constitute a rough simplification for some of the motivating applications, overcomes the indicated limitations. A related though more limited generalization of the SIS model can be found in the SISa model [36] (see [1, §X], for other generalizations of epidemic models as social contagion processes). The key assumption in our model is that individuals' state transitions depend on their current state and on the state of the individuals they interact with. We show that the qualitative results that we have described for the two-group SIS model, namely the existence of situations where the equilibrium diffusion levels are non-monotonic functions of the mixing level *m*, as well as the existence of Pareto-inefficient outcomes, hold in this more general case.

Consider an extended model which includes as parameters for each population i∈{1, 2} the following contagion and recovery rates, conditional on the current state of the interacting individual (state *S* or state *I*):

— $\upsilon_{i|S}$: rate at which individuals in state *S* who interact with individuals in state *S* adopt state *I*. In an infection model, this is the rate of infection when meeting a healthy partner (due to other factors of infection). In a school motivation model, this would be the rate at which students in group *i* in motivation state *S* and with a partner in motivation state *S* adopt motivation state *I* (due to other motivating factors).
— $\upsilon_{i|I}$: rate at which individuals in state *S* who interact with individuals in state *I* adopt state *I*. This is the rate of infection when meeting an infected partner.
— $\delta_{i|S}$: rate at which individuals in state *I* who interact with individuals in state *S* adopt state *S*. This is the rate of recovery when having a healthy partner.
— $\delta_{i|I}$: rate at which individuals in state *I* who interact with individuals in state *I* adopt state *S*. This is the rate of recovery when having an infected partner.

Leaving apart the limit cases in which some parameters are either 1 or null, we assume that $0<\upsilon_{i|S}<\upsilon_{i|I}<1$ and $0<\delta_{i|I}<\delta_{i|S}<1$.

The probability that an individual in group *i* interacts with an individual in state *I* is $I_{i}=(1-m)\rho_{i}+m\rho_{j}$, with index *j* indicating the other group: j∈{1, 2},j≠i.

The system of differential equations describing the evolution of adoption in each group over time is:

$${\dot{\rho }}_{i}=(1-\rho_{i})[\upsilon_{i|I}I_{i}+\upsilon_{i|S}(1-I_{i})]-\rho_{i}[\delta_{i|I}I_{i}+\delta_{i|S}(1-I_{i})]$$

To interpret these equations, note that $\left(1-\rho_{i}\right)$ is the prevalence of individuals in group *i* that can change to state *I* (because they are in state *S*), $\upsilon_{i|I}I_{i}$ is the rate at which such individuals meet a partner in state *I* and then change to state *I*, and $\upsilon_{i|S}(1-I_{i})$ is the rate at which such individuals meet a partner in state *S* and then change to state *I*. The rest of the terms can be interpreted similarly.

For each value of *m* and $\rho_{j}\in [0,\;1]$, ${\dot{\rho }}_{i}$ is a second-degree polynomial in *ρ_i_* such that ${\dot{\rho }}_{i}(\rho_{i}=0)=\upsilon_{i|S}+m\rho_{j}(\upsilon_{i|I}-\upsilon_{i|S})>0$, and ${\dot{\rho }}_{i}(\rho_{i}=1)=-[\delta_{i|I}+m(1-\rho_{j})(\delta_{i|S}-\delta_{i|I})]<0$.

Consequently, for each value of *m* and $\rho_{j}\in [0,\;1]$ there is a unique and positive value of $\rho_{i}\in [0,\;1]$ satisfying ${\dot{\rho }}_{i}=0$, and this equation defines a ‘reaction function’ $\rho_{i}^{R}:[0,\;1]\times [0,\;1]\to (0,\;1)$, $\left(\rho_{j},m)\to \rho_{i}^{R}(\rho_{j},m\right)$, which provides the corresponding equilibrium diffusion level in group *i*.

It is not difficult to see that, for a fixed value of *m*, $\rho_{i}^{R}$ is strictly increasing in *ρ_j_* (this follows from the fact that $\partial {\dot{\rho }}_{i}\text{/}\partial \rho_{j}>0$) and that, if $\left(\upsilon_{i|I}-\upsilon_{i|S})>(\delta_{i|S}-\delta_{i|I}\right)$, it is also strictly concave (strictly convex if the inequality is reversed). For the strictly concave–concave case, as well as for the strictly convex–convex case (which can be easily transformed to the concave–concave case via a change of variables), the same arguments that we used in §A2 of the electronic supplementary material for the SIS model can be applied to show that there is a unique interior globally asymptotically stable stationary state.

**Figure 3.**
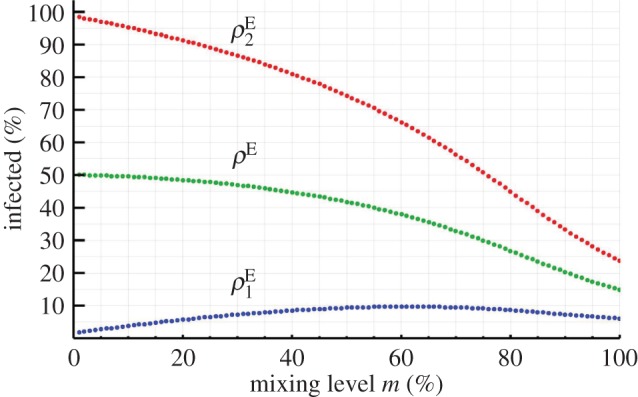
Equilibrium diffusion values as a function of the mixing level in the general contagion model. Parameter values (%): $(\upsilon_{1|S},\upsilon_{1|I},\delta_{1|I},\delta_{1|S})=(1,15,60,70);\;(\upsilon_{2|S},\upsilon_{2|I},\delta_{2|I},\delta_{2|S})=(1,80,1,20).$

Figure 3 illustrates the equilibrium diffusion values ($\rho_{1}^{E},\rho_{2}^{E}$) as a function of the mixing level *m* for a parametrization of this model. It shows that the same qualitative features that we discussed for the multi-group SIS model also apply to this extended case: the diffusion level in a group can be a non-monotonic function of the mixing level, and there can be Pareto-inefficient mixing levels.^9^ Again, for policy considerations, it also shows that the qualitative global effect of modifying the level of segregation or mixing between groups varies with the parameter values of the groups: given most reasonable sets of objectives, a modification of the mixing level in one direction (increase or decrease) could be beneficial or prejudicial depending on the status quo and on the parameter values of both groups. Particularly, for the issue of ability sorting in schools (for which empirical findings are often controversial and sometimes even contradictory [27–29]), our extended model would suggest a way of reconciling apparently opposing recommendations within one single coherent explanation: increasing the level of mixing between students with high and low academic performance can have a positive or negative overall effect depending on students' responsiveness to their partners’ state.

## 5. Discussion

Humans belong to different groups according to race, gender, age, abilities, preferences, etc. Most kinds of human interactions are biased in the sense that they take place preferentially between individuals of the same group (homophily) or, conversely, between individuals of different groups (disassortative mixing). Nonetheless, social policies can be implemented to modify the level of mixing between different groups. We have studied the effect on diffusion processes—such as the adoption of a particular behaviour or the spread of a disease—of mixing two groups with different propensities for adoption.

Our results using a SIS model show that, for some parameter regions, the diffusion level in the resistant group is a non-monotonic function of the mixing level *m* (proposition 3.3). This is a consequence of two opposing effects taking place simultaneously when increasing the mixing level from an initial equilibrium situation. On the one hand, by mixing more with the sensitive group, which has more infected individuals or adopters, the number of adopters in the resistant group should increase. On the other hand, a greater mixing with the resistant group decreases the diffusion level in the sensitive group (proposition 3.2). This, in turn, creates a feedback loop effect on the resistant group such that, when the reduction of adopters in the sensitive group is strong enough (i.e. when *m* is sufficiently high), the number of adopters in the resistant group also decreases as mixing increases.

If the diffusion level in the resistant group is non-monotonic, there are Pareto inefficient mixing levels, i.e. mixing levels such that both groups would be better off at some other mixing level. In this case, avoiding Pareto-dominated outcomes, which can be considered a generally desirable objective [2], would rule out a range of (non-efficient) mixing levels, though, in general, it would not provide just one single optimal mixing level. Optimizing the *average diffusion* is another possible objective for a social planner to focus on. In such a case, and depending on the groups' adoption rates, the optimal mixing level can range from the totally segregated case (*m* = 0) to the bipartite case (*m* = 1). An alternative objective worth exploring can be to reduce the diffusion difference between groups. To achieve this, the bipartite case where all interactions are between groups (*m* = 1) is always the best structure (proposition 3.5). However, it should be noted that this situation may be Pareto inefficient (see, e.g. the graph representative of region C in figure 2, assuming a desirable trait).

The generalization of the SIS model that we have considered also shows parameter regions such that the equilibrium diffusion levels are non-monotonic functions of the mixing level *m*, with Pareto-inefficient mixing levels.

To conclude, our results indicate that the best mixing level among heterogeneous groups will generally depend not only on the desired objective (such as, for instance, maximizing the average diffusion level), but typically on the effective adoption rates (or propensities) of each group, these being parameters that are well defined and which can be measured or estimated. A ‘one-fits-all’ recommendation does not exist, meaning that the optimal policy could be very different for different contexts. Furthermore, the existence of inefficient mixing levels highlights the importance of estimating appropriately the relevant contagion parameters before embracing any particular policy.

## Supplementary Material

Supplementary Material

## Footnotes

1 For extensions of epidemic models to networks, see [1,5–8].

2 Also related, there have been some reports of a non-monotonic effect of mixing on diffusion levels in simulation experiments [17]. More distantly related to our study, considering costly strategic decisions that can mitigate the infection process, two-type SIS models for different mixing levels have been studied with relation to the issue of strategic immunization or adoption of prevention measures [18,19].

3 To make statements about individuals’ well-being, first we have to assume that the infected state is either desirable or undesirable. If the infected state is undesirable, then each group's well-being decreases with its own equilibrium infection level, i.e. the fewer infected individuals, the better. In contrast, if the infected state is desirable, the group's well-being increases with its own infection level, i.e. the more infected individuals, the better. Notice that, in this framework, the equilibrium determines an average fraction of infected individuals in each group, but the identity of such individuals will typically change from one period to another: agents are permanently switching from infected to susceptible and vice versa.

4 Naturally, we do not claim that our simple model can be *directly* applied to any such situations and used to automatically derive policy recommendations in settings where other factors not included in the model may play an important role. Our model has been purposefully designed to illustrate a phenomenon that can occur in a variety of contexts and, precisely for this reason, it is highly stylized.

5 There is a close relationship between the mixing level in our model and the concept of modularity in general networks. The modularity of a partition of a network into groups is the fraction of intra-group links minus the expected value of such a fraction if links were distributed at random [30,31]. For networks with two groups where the number of links departing from nodes in each group (i.e. the sum of the degrees of all nodes belonging to each group) is the same, as in our model on expectation, the fraction of between-group links *m* and the modularity *Q* of the partition given by the groups are linearly related in a particularly simple way: *Q *= (1 – *m*) – ½ =½ – *m*. Similarly, the relationship between the mixing level *m* and the assortativity coefficient *r* [32] in our model is also straightforward: *r *= 1 – 2*m*.

6 Unless infection is desirable in one group and undesirable in the other, a case we do not consider here.

7 In these situations, for a resistant individual, and given some positive mixing level, coordinating with the other resistant individuals to increase the mixing level can be advantageous to reduce the average time infected, even though more mixing with susceptible individuals is individually costly if the other resistant individuals do not follow suit. In this sense, in a strategic setting, increasing the mixing level could constitute a social dilemma. However, it follows from proposition 3.3 that no situation for the resistant group can be strictly better than the situation with no mixing.

8 The behaviour in region B is somewhat less surprising once it is understood that enough mixing can eliminate the infection, and considering that a positive level of infection in one group implies a positive level of infection in the other. Continuity would then make the blue line in graph B go from a no-infection level at *m *= 0 back to a no-infection level again for sufficiently high mixing, going through positive values in the way.

9 There are also parameter combinations for which there are no inefficient mixing levels. For instance, for the ‘mirror’ case in which $\upsilon_{i|1}=\delta_{j|0}$ and $\upsilon_{i|0}=\delta_{j|1}$ , with i,j∈{1,2}, i ≠j, it can be easily shown that $\rho_{1}^{E}+\rho_{2}^{E}=\;1$, so any increase in one of the equilibrium infection levels must correspond to a decrease in the other.

## Data accessibility

A computer program available at https://luis-r-izquierdo.github.io/micopro/ implements the two-type SIS model used in the robustness analysis. An interactive Mathematica demonstration that computes and displays the equilibrium infection levels as a function of the mixing level is available at http://demonstrations.wolfram.com/MixingAndInfectionInATwoGroupSISModel/.
