# Supplementary material for: Mixing and diffusion in a two-type population
Source: R Soc Open Sci. 2018 Feb 21;5(2):172102. doi: 10.1098/rsos.172102 (PMC5830792; doi:10.1098/rsos.172102)
Supplement: Supplementary Material [file rsos172102supp1.docx]

Supplementary Material

Let *ρ_i_* denote the fraction of infected individuals in group *i*. The evolution of *ρ_i_* in each group over time is described by the following non-linear system of differential equations:

$\dot{\rho}_{i}=p\upsilon_{i}(1-\rho_{i})[m\rho_{j}+(1-m)\rho_{i}]-\delta_{i}\rho_{i}$ (1)

where $m$ is the mixing level, *i*, *j* ∈{1, 2} and *i* ≠ *j*. Note that parameters *p*, *υ_i_* and *δ_i_* can be interpreted as rates instead of probabilities. We can rewrite these equations as:

$\dot{\rho}_{i}=\delta_{i}[\lambda_{i}(1-\rho_{i})[m\rho_{j}+(1-m)\rho_{i}]-\rho_{i}]$ (2)

where *λ_i_* = (*p υ_i_* / *δ_i_*) represents the *effective adoption rate* *for group* *i.*

### A1. Stationary states for $\mathbf{m=0}$, $\mathbf{m=1}$ or $\boldsymbol{\lambda}_{\mathbf{1}}\mathbf{=}\boldsymbol{\lambda}_{\mathbf{2}}$

If *m* = 0, then each group is independent of the other and it is quick to check that the stationary states for group $i\in\left\{ 1,2 \right\}$ are $\rho_{i}=0$ and, if $\lambda_{i}>1$, also $\rho_{i}=1-\lambda_{i}^{-1}$.
If *m* = 1 the stationary states can also be found directly by solving $\dot{\rho_{1}}=0$ and $\dot{\rho_{2}}=0$, leading to $(\rho_{1},\rho_{2})=(0, 0)$ and, if $\lambda_{1}\lambda_{2}>1$, also $(\rho_{1},\rho_{2})=(\frac{\lambda_{1}\lambda_{2}-1}{\lambda_{1}\lambda_{2}+\lambda_{2}},\frac{\lambda_{1}\lambda_{2}-1}{\lambda_{1}\lambda_{2}+\lambda_{1}})$.
For $\lambda_{1}=\lambda_{2}$ the stationary states are $(\rho_{1},\rho_{2})=(0, 0)$ and, if $\lambda_{1}>1$, also $\rho_{1}=\rho_{2}=1-\lambda_{1}^{-1}$. In this case the stationary states are not affected by the value of the mixing level $m\in(0, 1]$.

### A2. Proof of Proposition 3.1

By substituting the values $\rho_{1}=0$ and $\rho_{2}=0$ in Eq. 1, it is quick to check that the no-diffusion state $(\rho_{1},\rho_{2})=(0, 0)$ is a stationary state. We will show that, under certain conditions, it is unique and globally asymptotically stable; otherwise, there is a unique positive stationary state which is almost globally asymptotically stable.

Apart from the no-diffusion state, we can restrict the analysis of stationary states to the region $(\rho_{1},\rho_{2})\in(0, 1)\times(0, 1)$: at any stationary state the diffusion levels in each group must be less than one (note in Eq. 1 that if $\rho_{1}=1$ then $\dot{\rho_{1}}<0$) and the only stationary state in which the equilibrium value in some group is null is the no-diffusion state (note in Eq. 1 that, for $m>0$, $\dot{\rho_{1}}=0$ with $\rho_{1}=0$ imply $\rho_{2}=0$).

For $\rho_{2}\in(0,1)$ and $m\in(0,1)$, $\dot{\rho_{1}}$ is a second-degree polynomial in $\rho_{1}$:

$$\dot{\rho_{1}}=\delta_{1}[-\lambda_{1}(1-m)\rho_{1}^{2}+(\lambda_{1}(1-m+m\rho_{2})-1)\rho_{1}+\lambda_{1}m\rho_{2}]$$

This polynomial is such that $\dot{\rho_{1}}(\rho_{1}=0)=\delta_{1}\lambda_{1}m\rho_{2}>0$ and $\dot{\rho_{1}}(\rho_{1}=1)=-\delta_{1}<0$. Consequently, there is a unique value $\rho_{1}^{*}\in(0,1)$ such that $\dot{\rho_{1}}(\rho_{1}=\rho_{1}^{*})=0$. Besides, $\dot{\rho_{1}}<0$ if $\rho_{1}\in(\rho_{1}^{*},1)$ and $\dot{\rho_{1}}>0$ if $\rho_{1}\in(0,\rho_{1}^{*})$. The equation $\dot{\rho_{1}}=0$ defines a "reaction" function $\rho_{1}^{R}:(0, 1)\times(0, 1)\to(0, 1)$, $(\rho_{2},m)\to\rho_{1}^{R}(\rho_{2},m)$, which provides the corresponding equilibrium diffusion level in the resistant group and which, for any fixed value of $m$, can be shown to be a continuous strictly increasing and strictly concave function of $\rho_{2}$. [See A3]

Likewise, $\dot{\rho_{2}}=0$ defines a reaction function $\rho_{2}^{R}:(0, 1)\times(0, 1)\to(0, 1)$, $(\rho_{1},m)\to\rho_{2}^{R}(\rho_{1},m)$, which, for any fixed value of $m$, is a strictly increasing and strictly concave function of $\rho_{2}$.

**Fig. A1.** Reaction curves which do not meet at the origin: $\lim_{\rho_{1}\to0^{+}} \rho_{2}^{R}>0$

For any fixed value of $m$, a positive stationary state of Eq. 1 is an interior crossing point of the graphs of $\rho_{1}^{R}$ and $\rho_{2}^{R}$ in the $\rho_{1}-\rho_{2}$ plane in $(0, 1)\times(0, 1)$ - See Figure A1 and Figure A2-. Since these two continuous functions can be easily extended to the compact $[0, 1]\times[0, 1]$ and are increasing and concave with respect to their first argument, there is a unique positive stationary state of Eq. 1 if and only if:

i) Either any of the graphs does not converge to the origin, which happens if $\lim_{\rho_{1}\to0^{+}} \rho_{2}^{R}>0$ (as illustrated in Figure A1), or, otherwise,

ii) The graph of $\rho_{2}^{R}$ is above the graph of $\rho_{1}^{R}$ near the origin, which happens if the product of the slopes near the origin is greater than one, i.e., if $\lim_{\rho_{1}\to0^{+}}\frac{\partial\rho_{2}^{R}}{\partial\rho_{1}}\lim_{\rho_{2}\to0^{+}}\frac{\partial\rho_{1}^{R}}{\partial\rho_{2}}>1$ (as illustrated in Figure A2).

**Fig A2**. Reaction curves with slopes at the origin such that, besides the crossing point at the origin, there is an interior crossing point in$[0,1]\times[0,1]$ : $\rho_{1}^{R}$ is above $\rho_{2}^{R}$ near the origin.

Almost global convergence of Eq. 1 to the positive stationary state when it exists, or, otherwise, global convergence of Eq. 1 to the no-diffusion state, follow from the analysis of the vector $(\dot{\rho_{1}},\dot{\rho_{2}})$ in the different sub-regions of $(0, 1)\times(0, 1)$ obtained from the graphs of $\rho_{1}^{R}$ and $\rho_{2}^{R}$, as indicated in Figure A3. Note that sub-region A in Figure A3 does not exist when there is no interior stationary state, and then there is global convergence to the origin.


$$\rho_{2}$$

A

D

$$\rho_{1}$$

C

$$\rho_{1}^{R}$$

B

$$\rho_{2}^{R}$$

Fig. A3. Phase portrait schema for Eq. 1. The arrows next to the letters labelling each region and over the reaction curves indicate the positive components of the vector field at the interior of each region and at the reaction curves.

Considering that

$$\lim_{\rho_{1}\to0^{+}} \rho_{2}^{R}(\rho_{1},m)=Max(0,\frac{\lambda_{2}(1-m)-1}{\lambda_{2}(1-m)}),$$

and, if $\lambda_{2}(1-m)<1$,

$$\lim_{\rho_{1}\to0^{+}}\frac{\partial\rho_{2}^{R}}{\partial\rho_{1}}\lim_{\rho_{2}\to0^{+}}\frac{\partial\rho_{1}^{R}}{\partial\rho_{2}}=\frac{\lambda_{1}m}{1-\lambda_{1}(1-m)}\frac{\lambda_{2}m}{1-\lambda_{s}(1-m)},$$

the conditions for the existence of the almost globally asymptotically stable interior stationary state are $\lambda_{2}(1-m)\geq1$ or $\frac{\lambda_{1}m}{1-\lambda_{1}(1-m)}\frac{\lambda_{2}m}{1-\lambda_{2}(1-m)}>1$, which are equivalent (See A4) to $\lambda_{2}>1$ and either $\lambda_{1}\lambda_{2}>1$ or $m<\overline{m}=1-\frac{1-\lambda_{1}\lambda_{2}}{\lambda_{2}+\lambda_{1}-2\lambda_{1}\lambda_{2}}$.

### A3. Strictly increasing and strictly concave reaction functions.

From Eq. (2), let $F_{i}\equiv\lambda_{i}(1-\rho_{i})[m\rho_{j}+(1-m)\rho_{i}]-\rho_{i}$

Note that$\dot{\rho}_{i}=\delta_{i}F_{i}$. Solving $F_{1}=0$ for $(\rho_{1},\rho_{2})\in(0, 1)\times(0, 1)$ and $m\in(0, 1)$ provides the solution function

$$\rho_{1}^{R}=\frac{\lambda_{1}(1-m+m\rho_{2})-1+\sqrt{(\lambda_{1}(1-m+m\rho_{2})-1)^{2}+4\lambda_{1}^{2}m(1-m)\rho_{2}}}{2\lambda_{1}(1-m)}$$

An implicit differentiation of $F_{1}=0$ leads to

$$\frac{\partial\rho_{1}^{R}}{\partial\rho_{2}}=-\frac{\partial F_{1}/\partial\rho_{2}}{\partial F_{1}/\partial\rho_{1}}=\frac{\lambda_{1}m(1-\rho_{1})}{\lambda_{1}[m\rho_{2}+(1-m)\rho_{1}-(1-m)(1-\rho_{1})]+1}$$

which, considering $F_{1}=0$, simplifies to

$$\frac{\partial\rho_{1}^{R}}{\partial\rho_{2}}=\frac{\lambda_{1}m(1-\rho_{1})^{2}}{1-\lambda_{1}(1-m)(1-\rho_{1})^{2}}$$

Taking into account $F_{1}=0$ again, the term $\lambda_{1}(1-m)(1-\rho_{1})^{2}$ in the denominator is

$$\lambda_{1}(1-m)(1-\rho_{1})^{2}=\frac{(1-m)\rho_{1}}{m\rho_{2}+(1-m)\rho_{1}}(1-\rho_{1})<1$$

Consequently, $1-\lambda_{1}(1-m)(1-\rho_{1})^{2}>0$ and $\frac{\partial\rho_{1}^{R}}{\partial\rho_{2}}>0$.

To show that $\frac{\partial^{2}\rho_{1}^{R}}{\partial\rho_{2}^{2}}<0$ note that the function $\frac{\partial\rho_{1}^{R}}{\partial\rho_{2}}=\frac{\lambda_{1}m(1-\rho_{1})^{2}}{1-\lambda_{1}(1-m)(1-\rho_{1})^{2}}$ is increasing with respect to $(1-\rho_{1})^{2}$, while $\frac{\partial(1-\rho_{1}^{R})^{2}}{\partial\rho_{2}}<0$.

The proof for $\frac{\partial\rho_{2}^{R}}{\partial\rho_{1}}>0$ and $\frac{\partial^{2}\rho_{2}^{R}}{\partial\rho_{1}^{2}}<0$ proceeds analogously.

### A4. Existence regions for the interior almost globally asymptotically stable stationary state.

For $m\in(0, 1)$ and $0<\lambda_{1}<\lambda_{2}$ , the region satisfying

|  | $\lambda_{2}(1-m)\geq1$ | (3) |
| --- | --- | --- |
| or | $\frac{\lambda_{1}m}{1-\lambda_{1}(1-m)}\frac{\lambda_{2}m}{1-\lambda_{2}(1-m)}>1$ | (4) |

is the same region satisfying

|  | $\lambda_{1}\lambda_{2}>1$ | (5) |
| --- | --- | --- |
| or | $\lambda_{2}>1\mathrm{and}m<\overline{m}=1-\frac{1-\lambda_{1}\lambda_{2}}{\lambda_{2}+\lambda_{1}-2\lambda_{1}\lambda_{2}}.$ | (6) |

We will need some auxiliary results to prove this result. Note that, for $m\in(0, 1)$ and $0<\lambda_{1}<\lambda_{2}$ :

i) Any of the equations from Eq. 3 to Eq. 6 independently imply $\lambda_{2}>1$.

ii) For $\lambda_{2}>1$ and $\lambda_{1}\lambda_{2}\leq1$, Eq. 4 $\Longleftrightarrow$ Eq. 6. It is quick to check that, for $\lambda_{2}+\lambda_{1}-2\lambda_{1}\lambda_{2}>0$, Eq. 4 and Eq. 6 are the same condition. Besides, $\lambda_{1}\lambda_{2}\leq1$ implies $\lambda_{2}+\lambda_{1}-2\lambda_{1}\lambda_{2}>0$, given that $\lambda_{2}+\lambda_{1}-2\lambda_{1}\lambda_{2}\geq\lambda_{2}+\lambda_{1}-2\sqrt{\lambda_{2}\lambda_{1}}=(\sqrt{\lambda_{2}}-\sqrt{\lambda_{1}})^{2}>0$.

iii) If $\lambda_{2}>1$ and $\lambda_{1}\lambda_{2}\leq1$ then $0\leq\frac{1-\lambda_{2}\lambda_{1}}{\lambda_{2}+\lambda_{1}-2\lambda_{1}\lambda_{2}}<\frac{1}{\lambda_{2}}$ and, consequently, $\overline{m}>1-\frac{1}{\lambda_{2}}$ and Eq. 3 $\Longrightarrow$ Eq. 6.

Here we have used the result $\lambda_{2}+\lambda_{1}-2\lambda_{1}\lambda_{2}>\lambda_{2}(1-\lambda_{1}\lambda_{2}).$ To show this, note that $\lambda_{2}>1(\lambda_{2}-1>0)1-2\lambda_{2}>-\lambda_{2}^{2}$. Consequently,

$\lambda_{2}+\lambda_{1}-2\lambda_{1}\lambda_{2}=\lambda_{2}+\lambda_{1}(1-2\lambda_{2})>\lambda_{2}-\lambda_{1}\lambda_{2}^{2}=\lambda_{2}(1-\lambda_{1}\lambda_{2}).$

Proof of the equivalence of regions:

Eq. 3$\Longrightarrow$ (Eq. 5 or Eq. 6). Suppose Eq. 3 holds. Then either Eq. 5 or $\lambda_{1}\lambda_{2}\leq1$ with $\lambda_{2}>1$, in which case, because of iii), we obtain Eq. 6.

Eq. 4 $\Longrightarrow$ (Eq. 5 or Eq. 6). Suppose Eq. 4 holds. Then either Eq. 5 or $\lambda_{1}\lambda_{2}\leq1$ with $\lambda_{2}>1$, in which case, because of ii), we obtain Eq. 6.

Eq. 5 $\Longrightarrow$ (Eq. 3 or Eq. 4). Suppose Eq. 5 holds. Then either Eq. 3 or $\lambda_{2}(1-m)<1$, in which case Eq. 4 can be seen to be equivalent to

$$\lambda_{1}>\frac{1-\lambda_{2}(1-m)}{\lambda_{2}+(1-m)(1-2\lambda_{2})}$$

which holds because, for $\lambda_{2}>1$, we have

$$\frac{1-\lambda_{2}(1-m)}{\lambda_{2}+(1-m)(1-2\lambda_{2})}<\frac{1}{\lambda_{2}},$$

given that $\lambda_{2}+(1-m)(1-2\lambda_{2})>\lambda_{2}-(1-m)\lambda_{2}^{2}>0.$

Eq. 6 $\Longrightarrow$ (Eq. 3 or Eq. 4). Suppose Eq. 6 holds. Then either Eq. 5, in which case we have (Eq. 3 or Eq. 4), or $\lambda_{1}\lambda_{2}\leq1$, in which case, because of ii), we obtain Eq. 4.

### A5. Lemma 1

*Any stationary state* $(\rho_{1},\rho_{2})\neq(0, 0)$ *of Eq. 1 satisfies* $0<\rho_{1}<\rho_{2}<1$*. Furthermore,* $1-\lambda_{1}^{-1}<\rho_{1}<\rho_{2}<1-\lambda_{2}^{-1}$*.*

Proof: It was shown before that $0<\rho_{1}<1$ and $0<\rho_{2}<1$. Then, from $F_{1}=0$ and $F_{2}=0$ we obtain

$$\lambda_{1}<\lambda_{2}\Rightarrow\frac{\rho_{1}}{(1-\rho_{1})[m\rho_{2}+(1-m)\rho_{1}]}<\frac{\rho_{2}}{(1-\rho_{2})[m\rho_{1}+(1-m)\rho_{2}]}\Rightarrow$$

$$\Rightarrow\frac{\rho_{1}^{2}}{m(1-\rho_{1})+(1-m)\rho_{1}}<\frac{\rho_{2}^{2}}{m(1-\rho_{2})+(1-m)\rho_{2}}{\Rightarrow\rho}_{1}<\rho_{2}$$

where the last step follows from the fact that the function $f(x)=\frac{x^{2}}{m(1-x)+(1-m)x}$ is strictly increasing for $x>0$.

Now, given that the reaction functions are increasing in their first argument, we have that, at a stationary state $(\rho_{1},\rho_{2})$,

$$\rho_{1}=\rho_{1}^{R}(\rho_{2},m)>\rho_{1}^{R}(\rho_{1},m)=1-\lambda_{1}^{-1}$$

$$\rho_{2}=\rho_{2}^{R}(\rho_{1},m)<\rho_{2}^{R}(\rho_{2},m)=1-\lambda_{2}^{-1}$$

### A6. Derivatives of $\boldsymbol{\rho}_{\mathbf{1}}^{\mathbf{E}}$ and $\boldsymbol{\rho}_{\mathbf{2}}^{\mathbf{E}}$ with respect to $\mathbf{m}$.

These derivatives are defined in the region $\lambda_{2}>1$, with $m\in(0, 1)$ if $\lambda_{1}\lambda_{2}>1$, and $m\in(0, \overline{m})$ if $\lambda_{1}\lambda_{2}\leq1$.

Let $|J_{F,\rho}|$ be the determinant of the Jacobian matrix

$$J_{F,\rho}=\left( \begin{matrix} \partial F_{1}/\partial\rho_{1} & \partial F_{1}/\partial\rho_{2} \\ \partial F_{2}/\partial\rho_{1} & \partial F_{2}/\partial\rho_{2} \end{matrix} \right)$$

By the implicit function theorem, we have that, at any stationary point with $|J_{F,\rho}|\neq0$,

$$\frac{\partial\rho_{1}^{E}}{\partial m}=|J_{F,\rho}|^{-1}(\frac{\partial F_{1}}{\partial\rho_{2}}\frac{\partial F_{2}}{\partial m}-\frac{\partial F_{2}}{\partial\rho_{2}}\frac{\partial F_{1}}{\partial m}),\mathrm{and}$$

$$\frac{\partial\rho_{2}^{E}}{\partial m}=|J_{F,\rho}|^{-1}(\frac{\partial F_{2}}{\partial\rho_{1}}\frac{\partial F_{1}}{\partial m}-\frac{\partial F_{1}}{\partial\rho_{1}}\frac{\partial F_{2}}{\partial m})$$

where

$$\frac{\partial F_{1}}{\partial\rho_{1}}=\lambda_{1}[(1-m)(1-2\rho_{1})-m\rho_{2}]-1,$$

$$\frac{\partial F_{1}}{\partial\rho_{2}}=\lambda_{1}(1-\rho_{1})m,\mathrm{and}$$

$$\frac{\partial F_{1}}{\partial m}=\lambda_{1}(1-\rho_{1})(\rho_{2}-\rho_{1}),$$

with equivalent equations for the derivatives of $F_{2}$. Considering that, at a stationary state, $F_{1}=0$ and $F_{2}=0$, we also have

$$\frac{\partial F_{1}}{\partial\rho_{1}}=\frac{\lambda_{1}(1-\rho_{1})^{2}(1-m)-1}{1-\rho_{1}},\mathrm{and}$$

$$\frac{\partial F_{2}}{\partial\rho_{2}}=\frac{\lambda_{2}(1-\rho_{2})^{2}(1-m)-1}{1-\rho_{2}}$$

Leading to

$$\frac{\partial\rho_{1}^{E}}{\partial m}=|J_{F,\rho}|^{-1}\lambda_{1}(1-\rho_{1})(\rho_{2}-\rho_{1})[-m\lambda_{2}(1-\rho_{2})-\frac{\lambda_{2}(1-\rho_{2})^{2}(1-m)-1}{1-\rho_{2}}],\mathrm{and}$$

$$\frac{\partial\rho_{2}^{E}}{\partial m}=|J_{F,\rho}|^{-1}\lambda_{2}(1-\rho_{2})(\rho_{2}-\rho_{1})[m\lambda_{1}(1-\rho_{1})+\frac{\lambda_{1}(1-\rho_{1})^{2}(1-m)-1}{1-\rho_{1}}]$$

with

$$|J_{F,\rho}|=\frac{\lambda_{1}(1-\rho_{1})^{2}(1-m)-1}{1-\rho_{1}}\frac{\lambda_{2}(1-\rho_{2})^{2}(1-m)-1}{1-\rho_{2}}-\lambda_{1}\lambda_{2}m^{2}(1-\rho_{1})(1-\rho_{2})$$

Let us first show that $|J_{F,\rho}|\neq0$ at the positive stationary state.

As shown in B3, the first term in $|J_{F,\rho}|$ is not null at a stationary state, so

$$|J_{F,\rho}|=0\Rightarrow1-\frac{\lambda_{1}m(1-\rho_{1})^{2}}{1-\lambda_{1}(1-m)(1-\rho_{1})^{2}} \frac{\lambda_{2}m(1-\rho_{2})^{2}}{1-\lambda_{2}(1-m)(1-\rho_{2})^{2}}=0$$

But the product of the fractions in this expression is the product of the slopes of the reaction curves $\frac{\partial\rho_{1}^{R}}{\partial\rho_{2}}\frac{\partial\rho_{2}^{R}}{\partial\rho_{1}}$ which, following our previous discussion, must be less than one and positive at an interior crossing point. Consequently, $|J_{F,\rho}|>0$ at the positive stationary state.

### A7. Proof of Proposition 3.2

Solving $\frac{\partial\rho_{2}^{E}}{\partial m}=0$ with $F_{1}=0$ and $F_{2}=0$ provides a single solution at $m=\frac{\rho_{1}^{2}}{\rho_{1}-\rho_{2}}$ which, by lemma 1, is negative, so $\frac{\partial\rho_{2}^{E}}{\partial m}$ does not change sign for $m\in(0, 1)$. It is then quick to check that $\frac{d\rho_{2}^{E}}{dm}$ is negative in the region of existence of the positive stationary equilibrium, i.e., for $m\in(0, 1)$ if $\lambda_{1}\lambda_{2}>1$, or $m\in(0, \overline{m})$ if $\lambda_{1}\lambda_{2}\leq1$.

### A8. Proof of Proposition 3.3

Solving $\frac{\partial\rho_{1}^{E}}{\partial m}=0$ with $F_{1}=0$ and $F_{2}=0$ provides no solution for $m\in(0, 1)$ if $\lambda_{1}\geq\frac{\sqrt{\lambda_{2}}}{\lambda_{2}-\sqrt{\lambda_{2}}+1}$.
Given that $\frac{\sqrt{\lambda_{2}}}{\lambda_{2}-\sqrt{\lambda_{2}}+1}=\frac{\sqrt{\lambda_{2}}}{(\sqrt{\lambda_{2}}-1)^{2}+\sqrt{\lambda_{2}}}\leq1$, we have that if $\lambda_{1}>1$ then $\lambda_{1}>\frac{\sqrt{\lambda_{2}}}{\lambda_{2}-\sqrt{\lambda_{2}}+1}$. Besides, for $\lambda_{2}>1$, $\lambda_{1}>\frac{\sqrt{\lambda_{2}}}{\lambda_{2}-\sqrt{\lambda_{2}}+1}\Rightarrow\lambda_{2}\lambda_{1}>1$, and consequently the almost globally asymptotically stable positive equilibrium exists for $m\in[0, 1]$.
Considering that, if $\lambda_{1}>\frac{\sqrt{\lambda_{2}}}{\lambda_{2}-\sqrt{\lambda_{2}}+1}$, $\frac{d\rho_{1}^{E}}{dm}$ does not change sign with $m\in(0, 1)$, it is quick to check that $\frac{d\rho_{r}^{E}}{dm}$ is positive for $m\in(0, 1)$.

Solving $\frac{\partial\rho_{1}^{E}}{\partial m}=0$ with $F_{1}=0$ and $F_{2}=0$ provides, for $\lambda_{1}<\frac{\sqrt{\lambda_{2}}}{\lambda_{2}-\sqrt{\lambda_{2}}+1}$, a unique solution at $m=m_{1}\in(0,1)$ :

$$m_{1}=\frac{2\lambda_{1}\left( \sqrt{\lambda_{2}}-1 \right)^{2}}{\lambda_{1}+\lambda_{2}+2\lambda_{1}\lambda_{2}-4\lambda1\sqrt{\lambda_{2}}-\sqrt{4\lambda_{1}^{2}\lambda_{2}\left( \sqrt{\lambda_{2}}-1 \right)^{2}+\left( \lambda_{1}+\lambda_{2}-2\lambda_{1}\sqrt{\lambda_{2}} \right)^{2}}}$$

such that $\frac{d\rho_{1}^{E}}{dm}$ is positive for $m\in(0,m_{1})$ and negative for $m\in(m_{1},1)$ if $\lambda_{1}\lambda_{2}>1$, or for $m\in(m_{1},\overline{m})$ if $\lambda_{1}\lambda_{2}\leq1$.
The condition $\lambda_{1}<\frac{\sqrt{\lambda_{2}}}{\lambda_{2}-\sqrt{\lambda_{2}}+1}$ implies $\lambda_{1}<1$, so $\lim_{m\to0^{+}} \rho_{1}^{E}=0$.

### A9. Proof of Proposition 3.4

Solving $\frac{\partial\rho_{2}^{E}}{\partial m}+\frac{\partial\rho_{1}^{E}}{\partial m}=0$ leads to $\lambda_{1}(1-\rho_{1})^{2}=\lambda_{2}(1-\rho_{2})^{2}$. Together with $F_{1}=0$ and $F_{2}=0$, this provides the formula for the mixing level $m_{\rho}$that maximizes the average diffusion, from which the results follow:

$m_{\rho}=\frac{1}{2\lambda_{1}{(\lambda_{1}-\lambda_{2})}^{2}\lambda_{2}}\left[ 2\lambda_{1}^{2}\lambda_{2}+\lambda_{1}^{3}\lambda_{2}-4\lambda_{1}^{5/2}\lambda_{2}^{3/2}+2\lambda_{1}\lambda_{2}^{2}+6\lambda_{1}^{2}\lambda_{2}^{2}+2\lambda_{1}^{3}\lambda_{2}^{2}-4\lambda_{1}^{3/2}\lambda_{2}^{5/2}+\lambda_{1}\lambda_{2}^{3}+2\lambda_{1}^{2}\lambda_{2}^{3}-2\left( \lambda_{1}\lambda_{2} \right)^{3/2}-4\left( \lambda_{1}\lambda_{2} \right)^{5/2}-\sqrt{\lambda_{1}^{5}\lambda_{2}}-\sqrt{\lambda_{1}\lambda_{2}^{5}}+|\sqrt{\lambda_{1}\lambda_{2}}-1|\left( \sqrt{\lambda_{1}}-\sqrt{\lambda_{2}} \right)^{2}\sqrt{\lambda_{1}\lambda_{2}\left( \lambda_{2}^{2}+\lambda_{1}^{2}\left( 1+4\lambda_{2}^{2} \right)+2\lambda_{1}\lambda_{2}\left( 1-4\sqrt{\lambda_{1}\lambda_{2}} \right) \right)} \right]$

### A10. Proof of Proposition 3.5

Solving $\frac{\partial\rho_{2}^{E}}{\partial m}-\frac{\partial\rho_{1}^{E}}{\partial m}=0$ for $m$, with $F_{1}=0$ and $F_{2}=0$, provides a unique positive solution at $m=\frac{\rho_{2}\rho_{1}}{\rho_{2}+\rho_{1}-1}\frac{\rho_{2}+\rho_{1}-2\rho_{2}\rho_{1}}{(\rho_{2}-\rho_{1})^{2}}$. For $0<\rho_{i}<1$, this expression is greater than one: from expanding $(1-\rho_{1})(1-\rho_{2})>0$ it can be seen that the first fraction $\frac{\rho_{2}\rho_{1}}{\rho_{2}+\rho_{1}-1}$, if positive, is greater than one. The second fraction is greater than one because $\rho_{i}^{2}<\rho_{i}$. Consequently, $\frac{\partial\rho_{2}^{E}}{\partial m}-\frac{\partial\rho_{1}^{E}}{\partial m}$ does not change sign in the considered range of values of $m$ where the positive equilibrium exists. It is then quick to check that the difference is strictly decreasing.

### B. Robustness

A computer program available at <https://luis-r-izquierdo.github.io/micopro/> implements the two-type SIS model. It allows testing the validity of the mean-dynamic approximation for different population sizes and with heterogeneous propensities, uniformly distributed in each group around the group’s average value $\lambda_{i}$.

As an example, Fig. B1 shows the diffusion values obtained in each group and on average for a population of 1000 individuals with individual propensities in each group uniformly distributed in the range $\lambda_{i} \mp0.3 \lambda_{i}$, with $\lambda_{1}=0.25$for the resistant group, and $\lambda_{2}=2$for the sensitive group. The thick black lines correspond to the equilibria obtained with the mean dynamic equations. Each dot corresponds to an average across 10 independent samples of the time-averaged infection values obtained between time periods 10000 and 11000 (after the system has had time to evolve towards an equilibrium from the initial conditions).

**Fig. B1.** Average fraction of infected individuals in each group (red and blue dots) and in the whole population (green dots) as a function of the mixing level, with heterogeneous individual propensities within groups. The dots correspond to simulations of the process. The lines correspond to the equilibria obtained from the mean dynamic equations using the average values for the propensities in each group: $\lambda_{1}=0.25$ and $\lambda_{2}=2$. The dispersion range of individual propensities within each group is ±30% of the average value, following a uniform distribution in that range.

Even with the considered level of heterogeneity, the correspondence between the simulated values and the mean dynamic is very precise, except for the cases in which the expected equilibrium levels are positive but low (corresponding to mixing levels between 0.45 and 0.60 in the represented case), as some simulations will then reach the absorbing state where there is no infection and will remain there. In every other case, we obtain dispersion ranges between samples which are less than 1% for the resistant group and less than 6% for the sensitive group. The initial conditions turn out not to be relevant (as expected from our analysis), provided that the initial fractions of infected individuals are not too close to 0. The results in the graph were obtained with a 10% initial fraction of infected individuals.
